# Editorial: The 3 S's: sex, stress, and sleep as risk factors for dementias

**DOI:** 10.3389/fnagi.2024.1410797

**Published:** 2024-04-22

**Authors:** Qunxing Ding

**Affiliations:** Kent State University, East Liverpool, OH, United States

**Keywords:** gender, sleep, dementia, risk factor, neurodegeneration

This Research Topic delves into emerging factors beyond age that may influence the onset and severity of dementia. While age remains a primary factor, recent evidence suggests sex, stress, and sleep play significant roles. Sex differences in disease risk have sparked debate, yet studies indicate varying cognitive decline rates between genders. Stress, whether triggered by lifestyle, environment, or internal factors, impacts overall health and may contribute to dementia development via allostatic load. Irregular sleep patterns, prevalent in aging populations, pose a potential causal link to dementia-related disorders. This research aims to explore and analyze the latest findings on these impactful factors in dementia manifestation and progression. Three articles presented data related with gender and stress in this issue: a comprehensive study on the impact of prolonged social isolation (SIS) during adulthood in female leads to significant enhancements in acquisition skills, it also results in a slight impairment in memory retention, without altering anxiety and cortisol levels (Popović et al.). Moreover, males may become more prone to inflammatory states, while females are predisposed to oxidative states due to SIS (Oliva et al.). In addition, prolonged social isolation integrates cognitive-behavioral performance with multifractal dynamics of physiological signals along with the accumulation of tau and Aβ proteins in early-onset neurodegenerative conditions like Alzheimer's disease (Cavieres). These findings underscore the critical role of social interactions in the wellbeing and overall performance of social animals, shedding light on novel avenues for further exploration in the field of neuroscience and behavioral psychology.

There is no data about sleep in this issue. However, sleep is increasingly recognized as a critical factor in overall health, with emerging research shedding light on its significant role in the risk of developing dementia. The quantity, quality, sleep pattern, lifestyle factors, and other pathological conditions all contribute to the risk of dementia.

For sleeping quantity, insufficient sleep has been linked to an elevated risk of dementia. Hahn et al. (2014) found that reduced sleep time was associated with a staggering 75% increase in the risk of all-cause dementia. Similarly, Sabia et al. (2021) revealed that short sleep duration in midlife is correlated with a heightened risk of late-onset dementia. However, it's not just inadequate sleep that poses a risk. Surprisingly, prolonged sleep duration, defined as combining nighttime sleep with daytime napping totaling 9 h or more, may also be associated with an increased risk of dementia (Benito-León et al., 2009).

The quality of sleep has emerged as a critical factor in dementia risk. Brachem et al. (2020) observed that poor sleep quality and difficulties initiating sleep were associated with incident mild cognitive impairment (MCI), a precursor to dementia. Addressing sleep disturbances and deficiencies has shown promise in mitigating the risk of incident dementia and all-cause mortality among older adults, as highlighted by Robbins et al. (2021). Notably, Lysen et al. (2020) suggested that poor sleep quality, rather than disruptions in the 24-h activity rhythm, was specifically linked to an increased risk of dementia. Additionally, the presence of inflammation may serve as a significant determinant in understanding the relationship between sleep disturbances and neurodegeneration (Baril et al., 2021).

Changes in sleep patterns have been identified as potential contributors to the development of dementia, highlighting the importance of monitoring sleep behaviors for early detection and intervention. For instance, Hahn et al. (2014) found that 28.5% of individuals experiencing alterations in their sleep patterns were diagnosed with all-cause dementia, with 22.0% developing Alzheimer's disease (AD) within 6 to 9 years after baseline assessment. Furthermore, specific sleep behaviors such as spending extended periods in bed and adopting early sleep timing have been linked to heightened dementia risk. Notably, Liu et al. (2022) found that these associations with greater cognitive decline were particularly evident among older individuals aged 60–74 years and among men.

Moreover, various lifestyle factors, including sleep duration, leisure-time physical activity, and screen-based sedentary behavior, exhibit individual associations with dementia risk in non-linear patterns (Huang et al., 2022). Poor sleep is intertwined with several risk factors for dementia, including hypertension, diabetes, obesity, and cardiovascular disease, each capable of independently elevating the risk of cognitive impairment and dementia (Leritz et al., 2011; Feinkohl et al., 2018).

To bolster the connection, it's noteworthy that individuals with dementia frequently experience sleep disturbances. As people age, the duration of sleep tends to decrease, particularly in those affected by Alzheimer's disease (AD). Notably, studies have observed poor sleep quality and insufficient sleep in individuals exhibiting preclinical signs of AD (Roh et al., 2014). Inadequate sleep or sleep disorders could hinder the brain's clearance process, potentially leading to the buildup of amyloid plaques in APP/PS1 mice lacking orexin that sleep deprivation or increased wakefulness through orexinergic neuron rescue heightened A beta pathology in the brain (Roh et al., 2014; Eide et al., 2021). Moreover, adjunctive prolonged-release melatonin therapy has shown promise in enhancing cognitive function and promoting sleep maintenance in AD patients, according to Wade et al. (2014). Similar to the effects observed with A beta, acute sleep deprivation has been found to elevate tau levels in mouse brain interstitial fluid (ISF) and human cerebrospinal fluid (CSF), while chronic sleep deprivation accelerates the propagation of tau protein aggregates within neural networks (Wang and Holtzman, 2020). Additionally, chronic sleep deprivation or sleep disorders can trigger heightened levels of inflammation, a phenomenon implicated in the development and progression of various neurodegenerative diseases, including dementia, as highlighted by Irwin and Vitiello (2019). Disruption of the body's internal clock, or circadian rhythms, stemming from irregular sleep patterns or conditions like sleep apnea, may exert adverse effects on brain health, as elucidated by Foster (2020). Notably, sleep deprivation or poor sleep quality can lead to neuronal damage and impaired synaptic plasticity, crucial processes for learning and memory, and the potential mechanisms linking sleep-related brain activity to synaptic structural remodeling post-experience (Alkadhi et al., 2013; Sun et al., 2020).

In conclusion, research indicates that the quantity, quality, sleep pattern, lifestyle factors, and other pathological conditions are associated with an increased risk of dementia. Sleep disturbances are common in individuals with dementia, suggesting a bidirectional relationship between sleep and neurodegeneration. Mechanisms linking sleep disturbances to dementia risk include impaired amyloid and tau clearance, inflammation, and disruptions in circadian rhythms, all of which contribute to neuronal damage and cognitive decline. Interventions targeting sleep quality and duration show promise in mitigating dementia risk and improving cognitive function. Further research into the intricate interplay between sleep and dementia pathogenesis is crucial for developing effective preventive and therapeutic strategies.

## Author contributions

QD: Writing – review & editing, Writing – original draft.
